# Effect of Application of Ultrafine Water Droplets on Water Content in the Stratum Corneum Layer in Excised Human Skin and on Barrier Function in a Three‐Dimensional Cultured Human Skin Model

**DOI:** 10.1111/srt.70223

**Published:** 2025-09-29

**Authors:** Hiroaki Todo, Moe Hirota, Madoka Kage, Yutaka Takagi, Ibuki Narita, Shinsuke Inoue, Yuki Tabata, Keiko Yokoyama, Shoko Itakura, Syuuhei Komatsu

**Affiliations:** ^1^ Laboratory of Pharmaceutics and Cosmeceutics Faculty of Pharmacy and Pharmaceutical Sciences Josai University Sakado Japan; ^2^ Laboratory of Dermatological Physiology Faculty of Pharmacy and Pharmaceutical Sciences Josai University Sakado Japan; ^3^ Value Chain Business Center New Business Incubation Laboratory AIR Business Promotion Department AISIN Corporation Kariya Aichi Japan

**Keywords:** barrier function, ceramide metabolism, trans‐epidermal water loss, ultrafine water droplets

## Abstract

**Background:**

Skincare using facial humidifiers is gaining attention and has become more popular in Japan as a beauty treatment. A novel type of face humidifier capable of generating ultrafine water droplets has been developed recently.

**Materials and Methods:**

The change in distribution of sprayed ultrafine water droplets in the stratum corneum of excised human skin over the application period was investigated using confocal Raman microscopy. In addition, changes in trans‐epidermal water loss (TEWL) value were evaluated after treatment with ultrafine water droplets using a three‐dimensional cultured human skin model (3D skin model). Furthermore, the effect of gene expression related to ceramide production in the skin and ceramide production were also evaluated using a 3D skin model by real‐time PCR and high‐performance thin‐layer chromatography, respectively.

**Results:**

A higher water content at the surface of the skin was confirmed over 45 min after the application. Furthermore, the TEWL value after the application of ultrafine water droplets was sharply decreased. Increased gene expression related to ceramide production was confirmed compared with the application of purified water, and increased levels of ceramide NS were observed.

**Conclusion:**

These results suggested that the application of ultrafine water droplets on the skin surface may be useful to maintain a physiologically good skin barrier condition.

## 1 Introduction

The stratum corneum (SC), the outermost layer of the skin, is 10–20‐µm thick and contains 20–30 wt% water [1]. The SC functions not only to prevent water loss from the body but also to prevent the penetration of external chemical substances [2]. The amount of water in the SC is related to skin transparency and also affects the flexibility and elasticity of the skin [3, 4]. Furthermore, it is well known that the water content in the SC layer decreases with age [5, 6, 7] with a decreasing lipid content in the skin, and this age‐related decrease in water content in the SC is associated with decreased skin permeation of water‐soluble active cosmetic ingredients, such as arbutin, ascorbic acid, kojic acid, potassium 4‐methoxysalicylate, niacinamide, and tranexamic acid [8]. Therefore, low SC water content may cause a decrease in skin concentrations of such ingredients due to reduced skin permeation, which may lead to weakened effectiveness.

Facial humidifiers are gaining attention as more time is spent at home, and such devices have recently become more popular in Japan [9]. Facial humidifiers are able to provide water droplets onto the skin surface, with one of the effects expected to be an increase in the amount of water content in the SC. Recently, a novel type of face humidifier using a copolymer poly(3,4‐ethylene dioxythiophene)‐poly(styrene sulfonate (PEDOT/PSS) layer has been developed [10]. The water droplet release mechanism has been reported previously [11], so it is described here briefly. Water supplied from the environment is absorbed onto water‐absorbable sulfonic acid–coated PEDOT/PSS. The absorbed water is detached smoothly by heating the 10‐nm‐thick PEDOT/PSS layer on a thermoconductive metallic layer. Unlike commonly used face humidifiers, such as steam‐, vaporization‐, hybrid‐, and ultrasonic‐types, this novel model requires no manual filling with water before use. The released water droplets have unique characteristics, such as maintaining skin moisturizing effects [1, 11] and a skin permeation enhancement effect on caffeine, a model of hydrophilic chemical [11]. In addition, the size of the generated droplets is approximately 1.4 nm, and the water droplets have a negative charge by adsorption of OH^−^ on the hydrophilic surface layer [12]. It has been reported that recovery of impaired barrier function of the skin was achieved by the application of negatively charged particles on the skin [10, 13]. Therefore, it was considered that ultrafine water droplets might affect the skin barrier function.

In the present study, we evaluated the efficacy of ultrafine water droplets on the permeability barrier function, accompanied with the changes in SC depth–water content profile. Furthermore, the changes in ceramide metabolism in the epidermis were analyzed by using three‐dimensional cultured human skin models.

## 2 Material and Methods

### 2.1 Materials

Frozen excised human skin was purchased from Biopredic International (Rennes, France) (53‐year‐old, Caucasian, female, abdominal skin, BMI 29). Commercially obtained excised human skin was used in this study. Although ethical approval was not required, donor‐informed consent was obtained by the supplier in compliance with applicable ethical regulations. Three‐dimensional cultured human epidermis model, LabCyte EPI‐MODEL12 6D (3D skin model), and assay medium were purchased from J‐TEC (Aichi, Japan). Ceramide NS was purchased from Matreya (Pleasant Gap, PA, USA). A Fast Gene RNA Basic kit was purchased from Nippon Genetics (Tokyo, Japan). PrimeScript RT reagent kit (Perfect Real Time), TB Green Premix Ex Taq (Tli RNaseH Plus), and RNase‐free water were purchased from Takara Bio Inc. (Shiga, Japan). Silica‐coated titanium dioxide (Si–TiO_2_: MT‐100WP, particle size: 15 nm) was provided by Tayca Corporation (Osaka, Japan). Other reagents and solvents were reagent grade or HPLC grade and used without further purification.

### 2.2 Application of Ultrafine Water Droplets

**FIGURE 1:**
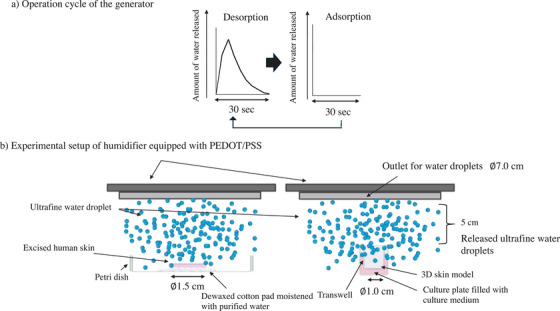
Adsorption and desorption cycle (a) and experimental setup (b) with a humidifier equipped with a PEDOT/PSS layer. The humidifier contains a PEDOT/PSS layer (10‐nm thickness) under a fan. The PEDOT/PSS layer absorbs water from the environment, and fine droplets are released by heating.

The generator of ultrafine water droplets was provided by Aisin Corp. (Aichi, Japan). The release mechanism of ultrafine water droplets was reported previously [11]. Briefly, ultrafine water was adsorbed at room temperature and dispatched by heating the PEDOT/PSS layers (up to 60°C). During the experiment, the operating cycle of the generator repeated water adsorption and desorption of ultrafine water droplets every 30 s. In the present study, water generator equipment with two release outlets was used, and the device was capable of releasing 15 µL of ultrafine water droplets in one cycle (1 min) of adsorption and desorption (Figure 1a).

When the generator of ultrafine water droplets was applied to excised human skin or the 3D skin model, the ultrafine water droplet outlet was set 5.0 cm from the SC surface and applied for 60 min. The experimental setup is shown in Figure 1b. When applying ultrafine droplets to the 3D skin model, plastic wrap was placed over the culture tray to prevent contamination by the ultrafine droplets onto the other 3D skin models. The ultrafine water droplets were emitted from the outlet of the generator with a diameter of 7 cm.

### 2.3 Observation of Water Distribution–Depth Profile in the SC

Change in water–depth profile in the SC after application of ultrafine water droplets was investigated with excised human skin (Ø: 1.5 cm, area: 1.77 cm^2^). Following thawing and careful removal of any excess water adhering to the surface of the SC side using a dewaxed cotton pad, the skin was placed with dermal side down in a plastic petri dish, which was covered with a thoroughly moistened dewaxed cotton pad with purified water. The ultrafine water droplet generator was operated for 60 min (amount of water sprayed over 60 min: 15 µL/min × 60 min, total 900 µL). As a control experiment, 900 µL of purified water was applied onto the skin instead of ultrafine water droplets for 60 min. Following wiping off of excess water from the skin surface, the water depth profile in the SC was investigated using an in vivo confocal Raman spectrometer (Gen2‐SCA, RiverD International B.V., Rotterdam, the Netherlands) for up to 45 min after the end of water application. In all measurements, the Gen2‐SCA probe was placed on the skin with a weight of approximately 5 g/1.77 cm^2^. Confocal Raman spectrometer measurements were performed using a 671‐nm laser at a resolution of 2/cm in the range of 2600–4000/cm at a depth of 0–10 µm from the skin surface. Measurements were taken at three to four points on each tissue sample.

### 2.4 Evaluation of Permeability of Barrier Function by Using 3D Skin Model

**FIGURE 2:**
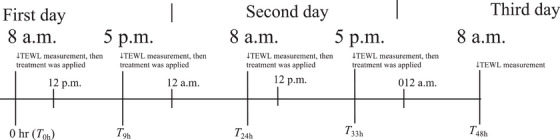
Time schedule of treatment of the 3D skin model and TEWL measurements.

The effect of the application of ultrafine water droplets on the permeability barrier function was evaluated using a 3D skin model by measuring trans‐epidermal water loss (TEWL). The skin model was incubated with culture medium containing 10 µL/mL of penicillin–streptomycin and 25 µg/mL of ascorbic acid at 37°C in 5% CO_2_, except during the application of ultrafine water droplets. The 3D skin model with culture medium was placed under the outlet of the ultrafine water generator at a distance of 5 cm, and ultrafine water droplets were applied to the 3D skin model for over 60 min in accordance with the schedule shown in Figure 2. Application of ultrafine water droplets for 60 min was performed twice a day for 2 days (at 8 a.m. and 5 p.m. on each day) after the TEWL measurement. A total of four treatments were applied; the first application was set at a cumulative time of 0 h (*T*_0h_), and the second, the third, and the final applications are expressed as *T*_9h_, *T*_24h_, and *T*_33h_, respectively. The 3D skin model was incubated at 37°C in 5% CO_2_ during the experiment, except during the measurement of TEWL and the application periods. As a control application, 900 µL of sterile water was applied to the 3D skin model. After the application of sterile water for 60 min, excess water on the 3D skin model was removed, and the same procedure for the application of ultrafine water was applied (treatment twice a day for 2 days, with daily application times of 8 a.m. and 5 p.m.). As a comparison, sterile water or a solution of 0.001% (w/v) Si–TiO_2_ suspended in sterile water was applied. TEWL was measured using a VAPO SCAN (AT‐VT100, Nihon Ash Co. Ltd., Tokyo, Japan). The 3D skin model was placed dermis‐side down onto a glass‐based dish so that water loss occurred only through the SC layer surface. Each measurement was repeated three times. The change in TEWL value (%) was calculated by subtracting the TEWL values at *T*_0h_ from the values at each measurement time (*T*_9h_, *T*_24h_, *T*_33h_, or *T*_48h_), and then the subtracted value was divided by the TEWL value at *T*_0h_ and multiplied by 100.

### 2.5 Efficacy of Ultrafine Water Droplets on Ceramide Metabolism

The 3D skin model with culture medium was placed under the outlet of the ultrafine water droplet generator at a distance of 5 cm, and ultrafine water droplets were applied to the 3D skin model for 60 min. Following application, the skin model was incubated with culture medium containing 10‐µL/mL penicillin–streptomycin and 25‐µg/mL ascorbic acid for 48 h at 37°C in 5% CO_2_. The cultured 3D skin model was washed three times on the SC and microporous membrane sides with phosphate‐buffered saline (PBS) and removed from the Transwell insert using a knife. RNA and lipids were extracted from the 3D skin model and analyzed as described below.

As a comparison, 0.001% (w/v) Si–TiO_2_ suspended in sterile water, which showed a zeta‐potential of −29.0 ± 0.231, measured using a Zetasizer Nano ZS (Malvern, Worcestershire, UK), was used to evaluate the efficacy of a negative charge on ceramide metabolism in the present study.

### 2.6 RNA Extraction and Quantitative Real‐Time PCR

Sterile water, 0.001% (w/v) Si–TiO_2_ suspended, and ultrafine water droplets were applied to the cultured 3D skin model for 48 h. The 3D skin model was collected from the culture insert and homogenized using a homogenizer pestle. Total RNA was extracted using a Fast Gene RNA Basic kit (Nippon Genetics, Japan) in accordance with the manufacturer's instructions. Total RNA concentration was measured using a Nanodrop 1000 (Thermo Fisher Scientific, DE, USA). cDNA was synthesized from 150‐ng total RNA by reverse transcription using a PrimeScript RT reagent kit and thermal cycler (Veriti; Applied Biosystems, CA, USA). Real‐time PCR analysis was accomplished using TB Green Premix Ex Taq (Tli RNaseH Plus) in 20‐µL final volumes. Amplification was performed using a StepOnePlus Real‐Time PCR System (Applied Biosystems). The sequences of the primers used were as follows: glyceraldehyde‐3‐phosphate dehydrogenase (GAPDH) 5′‐GAAGGTGAAGGTCGGAGT‐3′ (forward) and 5′‐GAAGATGGTGATGGGATTTC‐3′ (reverse); serine‐palmitoyl transferase‐1 (SPT‐1) 5′‐TGTTCCACCGTGACCACAAC‐3′ (forward) and 5′‐GCGCGCTACTTGGAGAAAGA‐3′ (reverse); serine‐palmitoyl transferase‐2 (SPT‐2) 5′‐CCTGCTCTTGTTGGCAAAGG‐3′ (forward) and 5′‐GCTCCCAGAACCAGTGATGC‐3′ (reverse); β‐glucocerebrosidase (β‐GCase) 5′‐GCTAGGCTCCTGGGATCGAG‐3′ (forward) and 5′‐GTTCAGGGCAAGGTTCCAGTC‐3′ (reverse); acidic sphingomyelinase (a‐SMase) 5′‐ACTTTGATAACTGCTCCTCTGAC‐3′ (forward) and 5′‐TTCGTGTCCAGCAGAGTACC‐3′ (reverse); fatty acid synthase (Fas) 5′‐GGCATCTGGACCCTCCTACCTCTG‐3′ (forward) and 5′‐CCTTGGAGTTGATGTCAGTCACTTGG‐3′ (reverse). The mRNA expression levels of *SPT‐1*, *SPT‐2*, *β‐GCase*, *a‐SMase*, and *Fas* were calculated using the 2^−ΔΔCt^ method by normalization relative to GAPDH mRNA. Results were expressed as an *n*‐fold difference relative to sterile water (relative expression levels).

### 2.7 Lipid Analysis

The removed 3D skin was soaked in 6 mL of chloroform:methanol (2:1, by vol) solvent and minced with scissors. The minced tissue was disrupted using an ultrasonic liquid processor (VCX 750, Sonics and Materials, CT, USA) at 750 W for 10 min on ice. Following filtration through a 0.2‐µm filter, the filtrate solvent was dried at 50°C under a stream of nitrogen gas and served as the lipid sample.

The dried samples were redissolved in 400‐µL chloroform:methanol (2:1, by vol) and analyzed using high‐performance thin‐layer chromatography (HPTLC). Extracted lipid samples (10 µL) and ceramide NS, which was used as a standard, were applied on a HPTLC silica gel 60 F_254_ plate (10 × 10 cm: Merck, Darmstadt, Germany). HPTLC plates were developed twice with chloroform:methanol:acetic acid (190:9:1, by vol). After the second development, lipids were visualized by treatment with 10% CuSO_4_, 8% H_3_PO_4_ aqueous solution, and heated to 180°C for 30 min using a TLC Plate Heater III (CAMAG, Muttenz, Switzerland). The amounts of extracted ceramides on the HPTLC plate were semi‐quantitatively analyzed using ImageJ analysis software (version 1.54 h).

### 2.8 Statistical Analysis

All experiments were repeated three to five times. Statistical significance of differences was examined for the results of water content–depth profile and change in the TEWL and using one‐way analysis of variance (ANOVA) followed by a Tukey–Kramer post hoc test. A paired Student's *t*‐test was carried out for the results of mRNA expression analyses.

## 3 Results

**FIGURE 3:**
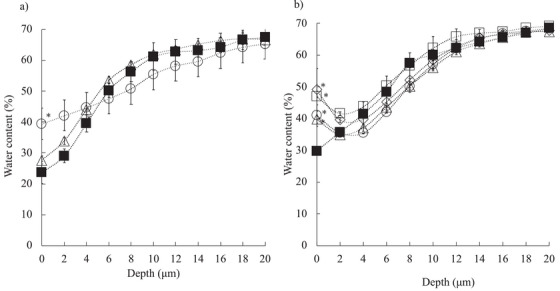
Change in the water content–depth profile in the SC layer after the application of purified water (a) and ultrafine water droplets (b). Symbols: ▪: before application, ○: just after application, □:15 min after the application, Δ: 30 min after the application, ⋄: 45 min after the application. Each experiment was repeated three to four times. Mean ± S.E. (*n* = 3–4), *p < 0.05 before application versus after application.

Prior to the application of purified water or ultrafine water droplets, the water content at the surface of the SC layer was approximately 30%, and at a depth of 12 µm, it was approximately 60% (Figures 3a and 3b). This water gradient in the SC layer was maintained over 5 h at room temperature (24 ± 3°C, 45 ± 5% RH) when the excised skin was placed on a dewaxed cotton pad moistened with purified water (data not shown).

After the application of purified water for 60 min, significant increases in water contents at depths of 0 and 2 µm were recorded; however, 30 min after application, these water contents returned to the initial levels (Figure 3a). On the other hand, the application of ultrafine water droplets resulted in the water content increasing significantly by approximately 40% at the surface in the SC layer, and this significant increase was maintained even after 45 min (Figure 3b).

Prior to water application, the water content was almost saturated at a skin depth of around 8–12‐µm depth at 60% concentration, which indicates that the thickness of SC was almost 12 µm. The water content at a depth of 12 µm decreased after the application of purified water for 60 min, indicating swelling of the SC. This swelling had almost recovered by 30 min after treatment. The application of ultrafine water droplets for 60 min also decreased the water content at a depth of 8 µm, but the difference at 10–12‐µm depth was lower, indicating that ultrafine water droplet treatment may induce less swelling of the SC.

**FIGURE 4:**
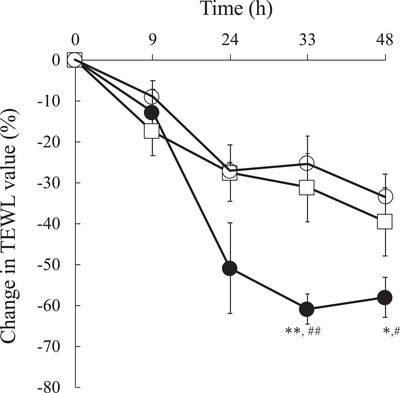
Change in TEWL value (%) in the 3D skin model. Symbols: ○: without application, □: sterile water application, •: ultrafine water droplet application. Each experiment was repeated three to four times. Mean ± S.E. (*n* = 3–4), * *p* < 0.05, **p < 0.01 without application versus ultrafine water droplets, #*p* < 0.05, ##*p* < 0.01 purified water versus ultrafine water droplets.

Figure 4 shows the changes in TEWL value in the 3D skin model following the application of purified water or ultrafine water droplets. The initial TEWL values for the control (without application), sterile water application, and ultrafine water droplet application were 26.8 ± 2.8 g/m^2^/h (mean ± S.E., *n* = 4), 26.5 ± 2.6 g/m^2^/h (*n* = 4), 26.7 ± 3.3 g/m^2^/h (*n* = 4), respectively. TEWL values decreased gradually in the control during the 48‐h incubation period. The application of purified water did not affect TEWL values by 48 h. On the other hand, the application of ultrafine water droplets decreased TEWL values at 24 h, and there was a significant difference compared with the other conditions at 33 and 48 h.

**FIGURE 5:**
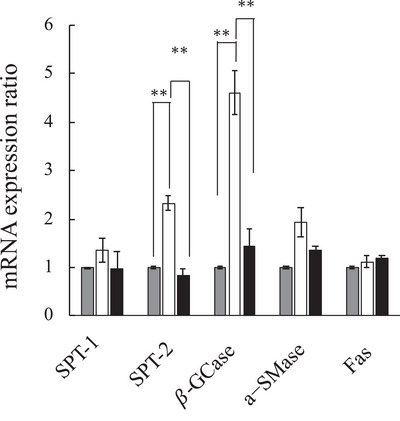
mRNA expression ratio after application of sterile water (gray filled color), 0.001% (w/v) Si–TiO_2_ suspended (black filed color) and ultrafine water droplets (white bar). mRNA levels were determined using quantitative real‐time PCR. Values were normalized to those after purified water application. Mean ± S.E. (*n* = 3–4), **p < 0.001: Si–TiO_2_ suspended versus ultrafine water droplet application.

Figure 5 shows gene expression levels related to ceramide metabolism in the 3D skin model 48 h after ultrafine water droplet treatment for 60 min. The gene expression levels of *SPT‐1*, *SPT‐2*, *β‐GCase*, *a‐SMase*, and *Fas* were 1.3‐, 2.3‐, 4.6‐, 1.9‐, and 1.1‐fold higher, respectively, compared with the application of sterile water. In particular, *SPT‐2* and *β‐GCase* exhibited significantly higher (*SPT‐2*: *p* < 0.001, *β‐GCase*: *p* < 0.05) expression levels than in the control. On the other hand, Si–TiO_2_ application, which was used as a comparison, did not induce increases in these gene expression levels. Accompanied by these increases in mRNA levels related to ceramide metabolism, ceramide levels in the 3D skin model obtained 48 h after the application of ultrafine water droplets were increased. Using semi‐quantitative densitometric analysis, ceramide NS levels were two‐fold higher (2.0 ± 0.25‐fold, mean ± S.E., *n* = 3) compared with the application of sterilized water. On the other hand, lipid profiles of ceramide AS and ceramide NP were hardly observed in the 3D skin model. A typical HPTLC result is shown in Figure S1.

## 4 Discussion

Nishimura et al. [1] reported the effect of sprayed particle size on skin conductance on the cheek, and water particle sizes below 0.5 µm generated using a PEDOT/PSS‐equipped device showed higher skin conductance 60 min after the application than application with a peak particle size of 1.8 µm. Furthermore, the TEWL value on the cheek after application of water particle sizes below 0.5 µm showed the lowest value compared with other larger particle sizes. In addition, higher skin conductance was confirmed after the application of the sprayed particles. They discussed that the smaller particle sizes of water droplets allowed them to penetrate deeper layers of the skin.

The skin surface is covered with ceramides, fatty acids, cholesterols, triglycerides, wax esters, and squalene [14]. They, therefore, consider the emulsification of fine water droplets with sebum components as a reason for preventing excessive water loss from the skin and showing high skin impedance. Todo et al. [11] also reported that ultrafine water droplets (about 1.4‐nm particle size) generated using a PEDOT/PSS‐equipped device exhibited a prolonged skin hydration effect after spraying compared with skin hydration with water and enhancement effect of the skin penetration of caffeine, a hydrophilic active ingredient of (molecular weight: 194 g/mol), due to hydration of the SC layer. Therefore, we considered that it is useful to clarify why the skin barrier function improves after the application of water droplets generated by PEDOT/PSS‐equipped devices.

In the present study, the water depth profile in the SC layer was investigated using freeze‐thawed human skin to clarify the characteristics of ultrafine water droplets. The SC structure after the thawing process might change compared with before freezing. However, excised freeze‐thawed human skin was used so that a constant adhesion force with the detector of the Raman microscope was maintained during the measurement period without moving. It was observed that higher water disposition on the skin surface was confirmed using Raman spectra observation over 30 min. Harada et al. [12] reported that ultrafine water droplets generated using a PEDOT/PSS‐equipped device formed ions (OH^−^) via surface adsorption from an uncharged state on a hydrophobic surface [HS‐(CH_2_)_11_‐(O‐CH_2_‐CH_2_)‐(CF_2_)_5_‐CF_3_ self‐assembled monolayer‐coated silicon carbide thin membrane]. This report suggested that because the skin is a hydrophobic membrane, ultrafine water droplets might adsorb onto the skin surface and become negatively charged. When purified water was applied, lower water content at greater depths in the SC layer was confirmed just after application, compared with the recordings before and 30 min after application. This decrease in water content was thought to be related to an increase in the thickness of the SC layer due to swelling caused by penetration of the applied water. This tendency, although slight, was also confirmed after the application of the ultrafine water droplets. This was thought to be due to the adsorption of the ultrafine water droplets on the skin surface.

Negatively charged particles of Si–TiO_2_ were used in the present study because it has been reported that negatively charged particles facilitated the repair of skin impedance caused by electroporation, which is a physical means to increase the skin permeation of poorly absorbable chemicals [13]. The skin surface is physiologically negatively charged, and the localization of calcium ions and their concentration gradient in the epidermis are essential in regulating many skin functions, including keratinocyte differentiation [15]. When skin is damaged, ion release occurs, inducing a voltage gradient across the damaged site [15], which activates signaling molecules involved in healing, such as integrins, epidermal growth factor receptors, and phosphoinositide 3‐kinases [16]. The application of charged materials that provide electrical fields on the skin might facilitate cell responses, including epithelial cells, fibroblasts, lymphocytes, macrophages, endothelial cells, and neuronal cells [17]. In addition, an electric field induced an increase in calcium ion levels in keratinocytes [17]. Extracellular calcium content and calcium influx into keratinocytes are related to the regulation of skin barrier recovery [15, 18]. Applying an external negative electric potential to human skin has already been investigated, and accelerated lamellar body secretion was confirmed compared with untreated control skin [18]. The lamellar body secretes precursors of ceramides, cholesterol, free fatty acids, and metabolic enzymes [19], and the application of ultrafine water droplets resulting in a negative charge on the skin surface may be a reason for the increase in gene expression levels related to ceramide and free fatty acid production.

The skin surface is physiologically negatively charged, and the localization of calcium ions and their concentration gradient in the epidermis are essential in regulating many skin functions, including keratinocyte differentiation. Negatively charged silica‐coated particles with a 15‐nm particle size were applied to the skin as a control to evaluate lipid production. On the other hand, the particle size of ultrafine water droplets was approximately 1.4 nm. Generally, a smaller particle size induces a stronger biological effect than larger particles, even though they have similar composition. Because surface reactivity might affect the physiological effects [20], further experiments should be conducted to examine the effect of the particle sizes of water droplets on skin hydration and SC barrier function.

A commercially available 3D skin model was used in the present study, instead of human skin, to evaluate lipid production. The lipid composition in commercially available 3D models (EpiDerm, SkinEthic, and Episkin) has been investigated [21]. Substantial differences were observed between 3D models and native human skin tissue, particularly polar ceramides, such as ceramide AH and ceramide AP, which might be involved in the higher permeability of chemicals through 3D skin models [22]. Although the lipid composition in 3D skin models used in the experiments was not compared with human skin, LabCyte Epimodel, which was the 2‐week incubated model used in the present study, exhibited faster permeation compared with excised human skin [23], suggesting that the contents of polar ceramides may be lower than in human skin tissue. The gene expression levels of ß‐GCase and a‐SMase were clearly increased, and the metabolite ceramides used by ß‐GCase are ceramide NS, ceramide NP, and ceramide AS, and those by a‐SMase are ceramide NS and ceramide AS [24]. The content of ceramide NS was increased, which might be considered the reason for the obvious increase in ceramide NS related to the characteristics of the 3D skin model. On the other hand, ceramide AS and ceramide NP were hardly observed in the 3D skin model, which might be attributable to the characteristics of the 3D skin model used in the present study. Further investigations should be performed on in vivo human skin to clarify the usefulness of the application of ultrafine water droplets.

Increased lipid production has been reported by supplementing the medium with ascorbic acid [25], but TEWL values, an indicator of reduced skin barrier function, decreased sharply after the application of ultrafine water droplets. The change in the TEWL values after 48 h of application of ultrafine water droplets was approximately −60%. This calculation suggests that the barrier function of the SC layer was increased by 2.5‐fold by accelerating SC layer maturation. The barrier function of the skin is regulated by the production of lipids. This result also suggested that the application of ultrafine water droplets improved the skin barrier by affecting the lipid production of ceramides.

In the present study, TEWL values after more than 48 h were not investigated, so it will be necessary to investigate whether TEWL values are affected over longer time periods. In addition, the water–depth profile in the 3D skin model might be different from that in human skin. Further experiments should be performed with human skin on the effect of the application of ultrafine water droplets on the change in the barrier function of the skin.

## 5 Conclusion

Water distribution in excised human skin and lipid production in a 3D skin model were investigated after the application of ultrafine water droplets generated using a PEDOT/PSS‐equipped device. The generated ultrafine water droplets were deposited on the skin surface with a longer‐term effect compared with purified water application, and increased gene expression related to ceramide production in the 3D skin model was confirmed. Subsequently, increased ceramide production and decreased TEWL values were obtained. Elucidating the characteristics of particles, including their stability on the skin surface, surface charge state, and changes in the lamellar structure induced by the interaction between the ultrafine particles and lipids, may help in understanding the effects on the skin induced by ultrafine water droplets. However, the obtained results suggested that the application of ultrafine water droplets on the skin surface, especially facial skin, may be useful to maintain a physiologically good skin barrier condition.

## Ethics Statement

This article does not contain any studies with human or animal subjects performed by any of the authors.

## Conflicts of Interest

Hiroaki Todo, Moe Hirota, Ibuki Narita, and Shuhei Komatsu have received a research grant from AISIN Corporation. Madoka Kage and Yutaka Takagi declare no conflicts of interest. Shinsuke Inoue, Yuki Tabata, and Keiko Yokoyama were employed by AISIN Corporation.

## Supporting information




**Figure S1**: HPTLC ceramide image of the 3D skin model after the application of ultrafine water droplets

## Data Availability

The data that support the findings of this study are available on request from the corresponding author. The data are not publicly available due to privacy or ethical restrictions.
