# Supplementary material for: Effect of Application of Ultrafine Water Droplets on Water Content in the Stratum Corneum Layer in Excised Human Skin and on Barrier Function in a Three‐Dimensional Cultured Human Skin Model
Source: Skin Res Technol. 2025 Sep 29;31(10):e70223. doi: 10.1111/srt.70223 (PMC12478874; doi:10.1111/srt.70223)
Supplement: Supplementary file 1 — Figure S1: HPTLC ceramide image of the 3D skin model after the application of ultrafine water droplets [file SRT-31-e70223-s001.pptx]

## Slide 1
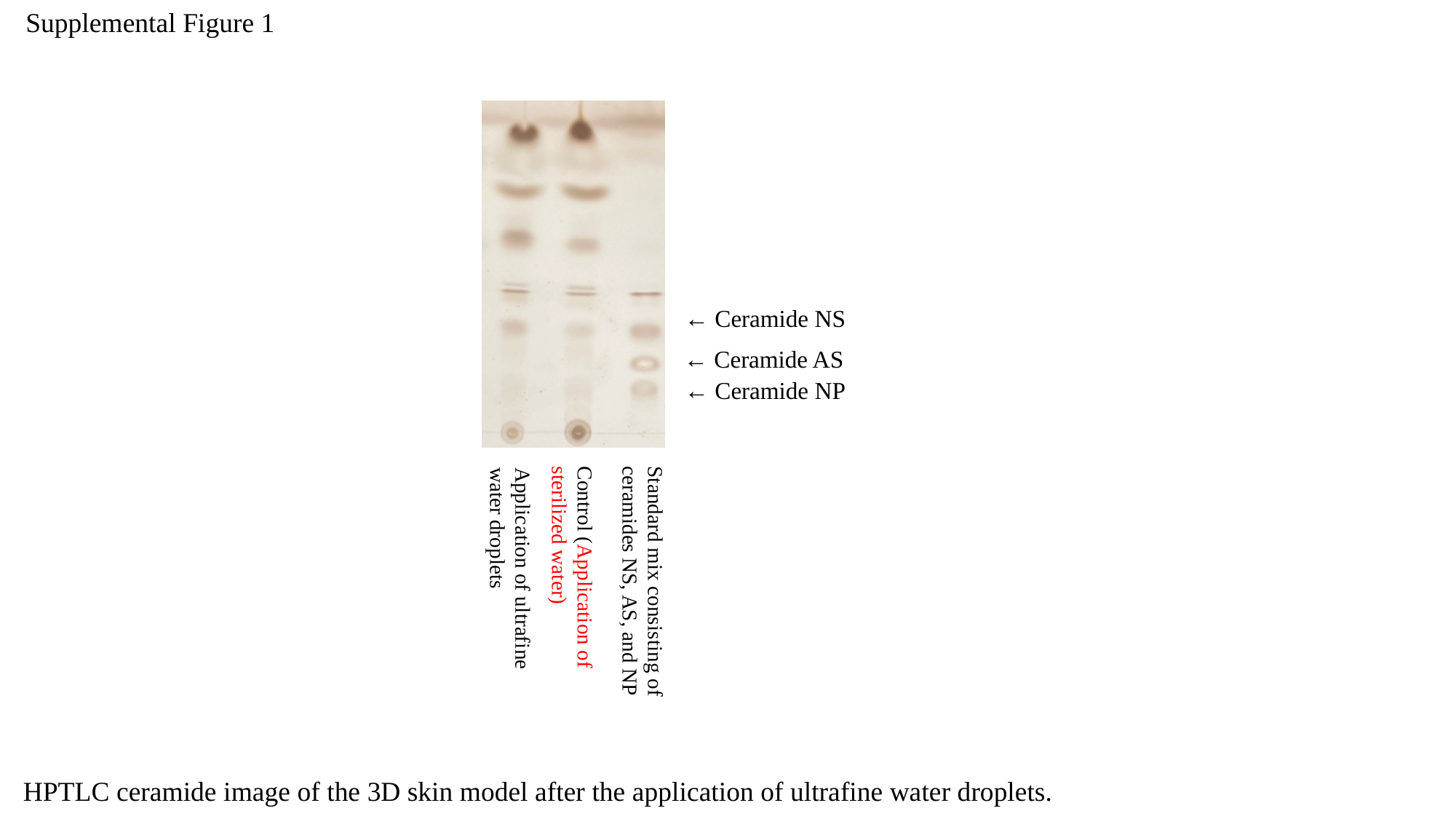

Supplemental Figure 1
← Ceramide NS
← Ceramide AS
← Ceramide NP
Control (Application of sterilized water)
Standard mix consisting of ceramides NS, AS, and NP
Application of ultrafine water droplets
HPTLC ceramide image of the 3D skin model after the application of ultrafine water droplets.
